# Case Report: Ensartinib as a first-line treatment for SMARCA4-deficient and EML4-ALK non-small cell lung cancer

**DOI:** 10.3389/fonc.2025.1530142

**Published:** 2025-05-09

**Authors:** Yanqing Pan, Lingxin Yan, Shaoxi Wang, Huiling Li, Quanfang Chen

**Affiliations:** ^1^ Department of Respiratory, The First Affiliated Hospital of Guangxi Medical University, Nanning, Guangxi, China; ^2^ Department of Respiratory and Critical Care Medicine, The First Affiliated Hospital of Guangxi Medical University, Nanning, Guangxi, China

**Keywords:** case report, ensartinib, SMARCA4-deficient, *EML4-ALK*, non-small cell lung cancer

## Abstract

SMARC4 is the catalytic subunit of the SWI/SNF chromatin remodeling complex and is one of the most common altered chromatin remodeling ATPases in cancer. Studies have indicated that SMARCA4 loss is associated with highly aggressive tumors, independently predicting shorter overall and disease-specific survival. SMARCA4-deficient non-small cell lung cancer (NSCLC) primarily affects male individuals, especially smokers, and is characterized by large, aggressive tumors. Cases of SMARCA4 deletion combined with actionable driver gene mutations (e.g., ALK) are rarely reported. In this report, we describe a male non-smoker diagnosed with SMARCA4-deficient, *EML4-ALK* non-small cell lung cancer who has been undergoing ensartinib targeted therapy for 3 months, resulting in a significant partial response. We also propose that, from a signaling perspective, the presence of SMARCA4 deficiency may influence the sensitivity of *EML4-ALK* NSCLC to targeted therapy, highlighting the need for further investigation into the underlying mechanisms and the exploration of novel therapeutic approaches.

## Introduction

SMARC4 is the catalytic subunit of the SWI/SNF chromatin remodeling complex and is located on chromosome 19p13.2 (1). The SWI/SNF complex plays a critical role in modulating chromatin structure and regulating gene expression, thereby maintaining normal cellular function. SMARCA4 is one of the most frequently altered chromatin remodeling ATPases in cancer, significantly impacting transcriptional regulation by disrupting histone–DNA interactions in an ATP-dependent manner (2). The deficiency of SMARCA4 impairs the function of the SWI/SNF complex, compromising chromatin regulation and increasing susceptibility to malignant transformation and tumor progression (3).

SMARCA4 mutations, regardless of mutation type, independently predict shorter overall and disease-specific survival (4, 5). SMARCA4-deficient tumors are typically high-grade malignancies that predominantly affect the chest, with a mutation rate of approximately 10% in non-small cell lung cancer (NSCLC) (6). The literature indicates that SMARCA4-deficient NSCLC primarily affects male individuals around the age of 60, especially smokers, and is characterized by large, aggressive tumors with high proliferation indices, evidenced by increased Ki-67 levels (7). Additionally, due to their genomic instability, frequent TP53 mutations, higher tumor mutations, and SMARCA4 deficiency, they react extremely poorly to treatment and have always been fatal within several months (8).

A notable feature of SMARCA4-deficient NSCLC is the low incidence of common targetable oncogenic mutations, such as ALK rearrangements (9). In this report, we reported the case of a 54-year-old man with SMARCA4-deficient NSCLC who also had *EML4-ALK*—a rare combination. The patient received ensartinib as a first-line targeted therapy for 3 months, resulting in significant tumor reduction without adverse effects. This case highlights the potential of ALK-targeted therapy in treating rare SMARCA4-deficient tumors and suggests a potential synergistic relationship between SMARCA4 deletion and ALK rearrangement in NSCLC.

## Case report

**Figure 1:**
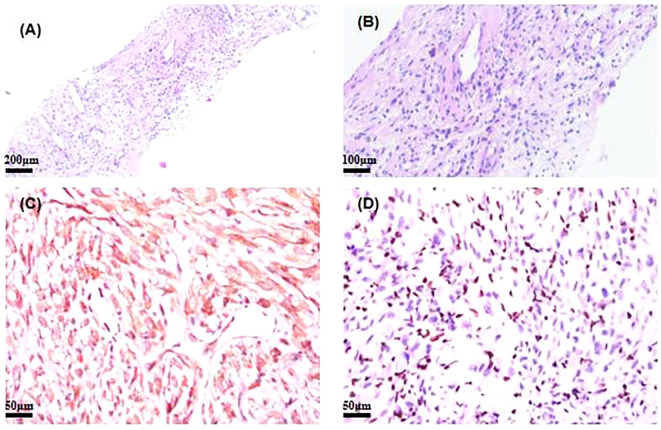
**(A, B)** Hematoxylin and eosin (H&E)-stained image of SMARCA4-deficient NSCLC. **(C)** Immunohistochemistry of ALK for SMARCA4-deficient NSCLC. **(D)** Immunohistochemistry of BRG1 for SMARCA4-deficient NSCLC. NSCLC, non-small cell lung cancer.

A 54-year-old man with no history of smoking, other malignancies, or relevant family history presented with a persistent dry cough and underwent chest computed tomography (CT) in June. The CT revealed a lesion in the left lung. When he presented at our hospital in July, a follow-up chest CT revealed a large, well-defined soft tissue mass adjacent to the left upper lobe, measuring approximately 11.9 cm × 8.4 cm × 13.1 cm (**Figure 1**), along with fluid in the left chest cavity; no enlarged lymph nodes were observed in the hilum or mediastinum. A whole-body bone scan showed no evidence of bone metastasis, and the brain CT was normal.

A core biopsy of the lung mass was performed under ultrasound guidance. Microscopically, the normal tissue structure was obscured, with abundant cytoplasm and atypical cells showing diffuse infiltration. The nuclei were round or irregular, with occasional visible nuclear grooves, and mitotic figures were rare. Histopathological examination showed poorly differentiated cancer. Immunohistochemical staining showed CK (+), ALK (+), EMA (−), CK8/18 (+), TTF−1 (−), P40 (−), Calponin (−), CD20 (−), and Ki-67 (+, 10%) (**Figure 1**). Next-generation sequencing of the tumor biopsy specimen detected an *EML4-ALK* gene fusion (abundance, 7.88%; average sequencing depth of target regions, 3,046.34) (**Table 1**). Due to the unusual histopathological features and atypical immunophenotype, we sent the slides to Shanghai Ackerman Biotechnology for further consultation. The results of the immunostaining showed that ALK immunohistochemistry was positive, while BRGI immunohistochemistry was negative (**Figure 1**). The final diagnosis was stage IV non-small lung cancer with SMARCA4-deficient and *EML4-ALK*, accompanied by pleural metastasis (staging was cT4N0M1a IVA).

**Table 1:** Next-generation sequencing results.

Pathogenic variant		
EML4-ALK	EML4 chr2: 42496614-ALK chr2: 29446757	VAF: 7.88%
Likely pathogenic variant		
TP53	Exon 5 c.481G>A p.A161T	VAF: 1.07%
Unknown significant		
CHEK2	Exon 5 c.613A>T p.T205S	VAF: 69.39%

VAF, Variant Allele Fraction.

**Figure 2:**
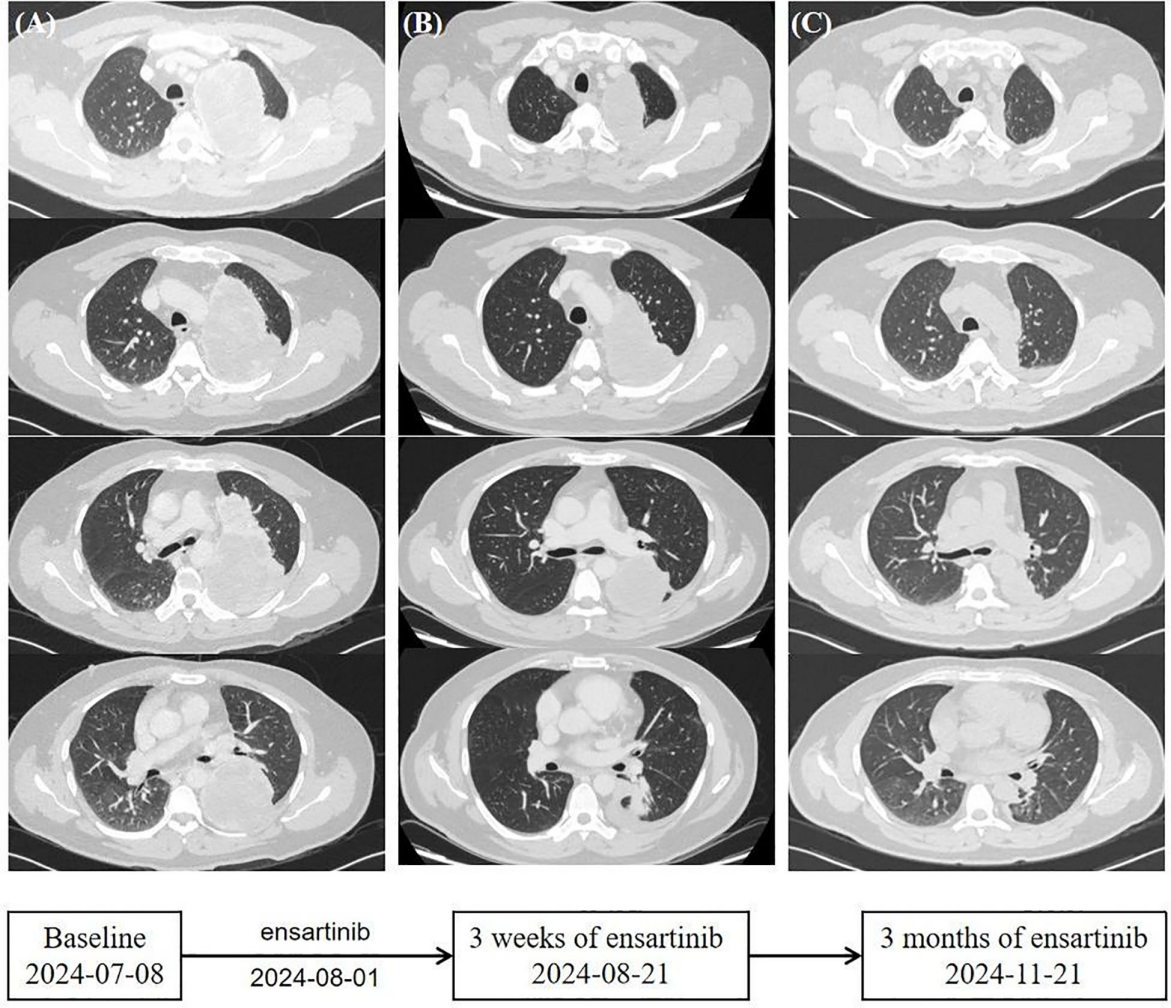
Timeline. **(A)** Computed tomography scans of the primary lung lesions at baseline before the initiation of ensartinib. **(B)** Ensartinib treatment for 3 weeks. **(C)** Ensartinib treatment for 3 months.

Based on guidelines, he began treatment with ensartinib (225 mg orally, once daily) in August after providing informed consent. After 3 weeks, the patient returned for a follow-up visit, and clinical evaluation showed that vital signs were stable. Blood tests indicated mildly increased liver enzyme levels, within the watchable range. Chest CT showed that the primary lung tumor lesion had reduced to 10.2 cm × 6.8 cm × 13.1 cm (**Figure 2**). Three months later, the patient underwent a second chest CT scan in the outpatient clinic for efficacy evaluation. The main tumor in the left lung had further regressed to approximately 3.4 cm × 4.4 cm × 8.4 cm (**Figure 2**). The pleural effusion was completely absorbed. No metastatic signs were observed in the contralateral lung. The sum of the target diameters decreased by 48% compared to the baseline, achieving Response Evaluation Criteria in Solid tumors (RECIST) 1.1 partial response (PR). No treatment-related adverse reactions were observed during the course of the medication. During home treatment, the patient was able to independently carry out daily tasks and activities, with no significant functional impairments or decline in quality of life. Based on the current clinical benefits, the patient and the clinician reached a consensus to continue the original treatment regimen. Subsequently, the patient will undergo regular follow-up as an outpatient.

## Discussion

In NSCLC, approximately 10% of cases exhibit SMARCA4 deletions (5). This alteration is associated with poor disease-specific survival and aggressive tumor behavior, which has garnered significant attention and is recognized as a distinct molecular subtype (10). SMARCA4-deficient NSCLC predominantly affects young and middle-aged male individuals with a history of smoking, often located in the mediastinal or hilar region, and usually presents with symptoms such as cough and sputum production (2). Diagnosis of SMARCA4-deficient NSCLC is primarily based on the loss of SMARCA4 protein expression, which can be detected through immunohistochemical staining for SMARCA4 (11). The concomitant loss of SMARCA4 in NSCLC presents a complex aspect of the pathogenic mechanisms underlying lung cancer. Structurally, SMARCA4 serves as the catalytic subunit of the SWI/SNF complex, playing a key role in the regulation of chromatin structure and gene expression. The loss of SMARCA4 enhances tumorigenesis by promoting genomic instability, thereby acting as a key driver of cancer development (8). Previous studies have indicated that SMARCA4 deficiency is associated with poor prognosis and reduced overall survival in NSCLC patients, as well as a higher likelihood of early postoperative recurrence and lower response rates to conventional treatments (4, 12). Additionally, SMARCA4 mutations are recognized as a genetic factor associated with poor clinical outcomes in lung cancer, regardless of whether patients undergo immunotherapy or non-immunotherapy treatments (13). In conventional chemotherapy, SMARCA4-deficient NSCLC has been found to exhibit sensitivity to platinum-based agents, particularly in patients with low BRG1 expression (14). Currently, NSCLC patients with oncogenic driver gene mutations show better outcomes with first-line targeted therapy compared to chemotherapy and anti-programmed cell death (anti-PD) immunotherapy (15). Fortunately, this patient exhibits *EML4-ALK* expression without widespread metastasis. Given the high invasiveness of SMARCA4-deficient NSCLC and the superior systemic and intracranial efficacy of ensartinib, ensartinib was selected as the first-line treatment (16). This case of SMARCA4-deficient NSCLC with *EML4-ALK* represents a rare combination. To date, only one case of SMARCA4-deficient NSCLC with concomitant ALK mutation has been documented in the global literature (17). The report described a 34-year-old woman diagnosed with an undifferentiated tumor exhibiting SMARCA4 deficiency, which demonstrated a fusion mutation involving EML4 exon 13 and ALK exon 20. Initial treatment with alectinib (600 mg, twice daily) resulted in a significant therapeutic response, as confirmed by a computed tomography scan 9 months later, showing complete remission. The difference was that this female patient was treated with alectinib after undergoing palliative thoracic surgery. Both patients with concurrent SMARCA4 deficiency and *EML4-ALK* demonstrated favorable responses following Tyrosine Kinase Inhibitor (TKI)-targeted therapy. We found that oral administration enhances patient adherence. Furthermore, our observations suggest that initiating treatment with the standard dose maximizes drug efficacy and optimizes the therapeutic response.

The mechanism is also worth thinking about. In malignant tumors, ALK mutations or chromosomal rearrangements result in the aberrant activation of ALK and its downstream signaling cascades, with the mitogen-activated protein kinase (MAPK) pathway serving as a critical downstream effector (18). The EML4-ALK proteins coordinate oncogenic signaling of the RAS/MAPK and JAK/STAT pathways by forming cytoplasmic compartments and recruiting proteins (19). MAPK is a signaling pathway that regulates cell growth, stress responses, differentiation, and viability (20). The MAPK signaling cascade enters the nucleus and has been shown to regulate post-transcriptional genes by interacting with transcription factors and chromatin remodeling enzymes, such as SWI/SNF (21). TKI-targeted inhibition of ALK can disrupt the *EML4-ALK* compartment, potentially interfering with the transcriptional regulation between MAPK and SWI/SNF, thereby affecting tumor cell proliferation (22). Therefore, we hypothesize that dysfunction of the SWI/SNF complex may have a potential impact on MAPK signaling, synergistically enhancing the anti-tumor effects of ALK inhibitors on tumor cell proliferation. However, this mechanism remains underexplored.

In summary, the interaction between ALK mutations and SMARCA4 deficiency in NSCLC highlights the complexity of lung cancer biology. Understanding the molecular mechanisms and clinical implications of these alterations is crucial for clinical treatment and improving patient prognosis. This case report presents an NSCLC with *EML4-ALK* and SMARCA4 deficiency, which showed a significant response to ensartinib. It provides a new reference for the treatment and research of SMARCA4-deficient NSCLC. However, the current number of cases is very small. Further research is needed to determine whether this approach can be broadly applied to other patients with similar genetic profiles.

## Data Availability

The original contributions presented in the study are included in the article/supplementary material. Further inquiries can be directed to the corresponding author.
